# CD133^+^ cancer stem cells promoted by VEGF accelerate the recurrence of hepatocellular carcinoma

**DOI:** 10.1038/srep41499

**Published:** 2017-01-30

**Authors:** Kai Liu, Meijun Hao, Yabo Ouyang, Jiasheng Zheng, Dexi Chen

**Affiliations:** 1 Capital Medical University affiliated Beijing You An Hospital, Beijing, 100069, China; 2 Beijing Institute of Hepatology, Beijing, 100069, China; 3 Organ Transplantation Center, the Affiliated Hospital of Qingdao University, Qingdao City, Shandong Province, 266003, China

## Abstract

The role of cancer stem cells (CSCs) in inducing the recurrence of hepatocellular carcinoma (HCC) after radiofrequency ablation (RFA) remains unclear. Here, we found that a dramatic increase in plasma vascular endothelial growth factor (VEGF) and an induction of local CD133^+^ CSCs are associated with early HCC recurrence, suggesting that VEGF expression and tumour stemness contribute to the relapse. *In vitro* studies demonstrated that VEGF, via activation of VEGFR2, increased the number of CD133^+^ CSCs and enhanced their capacity for self-renewal by inducing the expression of Nanog. *In vivo* studies further demonstrated that VEGF-treated CD133^+^ CSCs formed tumours larger than those developing from unstimulated cells and VEGF pre-treatment increased the tumorigenic cell frequency of primary HCC cells dependently on the presence of Nanog and VEGFR2. In HCC tissue derived from patients with early recurrence, almost all CD133^+^ cells were Nanog and p-VEGFR2 positive, suggesting that activation of VEGFR2 is critical for RFA-induced tumour stemness in HCC. In summary, RFA-induced VEGF promotes tumour stemness and accelerates tumourigenesis in HCC in a manner dependent on Nanog and VEGFR2, which is valuable for the prediction of HCC recurrence after RFA and the development of novel therapeutics.

Hepatocellular carcinoma (HCC) is one of the most lethal tumours worldwide. In China, HCC ranks as the second leading cause of tumour-related death^1^. Because resection is only applicable in a small number of cases, local destructive therapies, such as radiofrequency ablation (RFA), are vital alternative treatments. Although RFA shows comparable survival rates in some liver tumours, tumour recurrence can still occur^2^. To date, numerous studies have focused on describing the rate of recurrence after RFA; however, the mechanisms underlying recurrence remain unknown. The idea that cancer stem cells (CSCs) contribute to HCC development is suggested by data obtained from hepatectomy-/liver transplantation-related studies^3^. However, the effect of RFA on CSCs development is still unclear in HCC and other cancers.

CSCs are a small subpopulation of cancer cells with self-renewal and pluripotency abilities. CSCs-induced carcinogenesis models suggest that cancers arise and are maintained by this rare fraction of cells^4^. In many types of cancer, the existence of CSCs has been proven^5^. For example, HCC is organized as a hierarchy that originates from a primitive stem group of cells for which CD133^+^ precursors constitute one of the most immature stages^6^,^7^. CD133 (also known as Prominin 1) is a pentaspan transmembrane glycoprotein used in an increasing number of studies to identify CSCs in HCC^8^. CD133^+^ cells isolated from hepatoma cell lines and liver cancers possess greater colony-forming efficiency, higher proliferative output and an enhanced ability to form tumours *in vivo*^6^. In both HCC tumour tissue and adjacent non-cancerous liver tissue, CD133^+^ CSCs can be detected^9^. Because many studies have demonstrated that CSCs are responsible for the poor prognosis of patients by promoting tumour recurrence and metastasis^10^, it is rational to study the role of CD133^+^ CSCs in inducing HCC recurrence and some CSCs-related transcriptional factors (e.g., Nanog), since they regulate the self-renewal and differentiation of CSCs^11^.

Vascular endothelial growth factor (VEGF), one of the most potent angiogenic factors through activating VEGF receptor-2 (VEGFR2), is critical for the induction of microscopic venous invasion and metastasis in HCC^12^. VEGF was recently shown to regulate the initiation and stemness of skin tumours, indicating an important function of VEGF in the promotion and maintenance of tumour growth through the promotion of cancer stemness^13^. However, the exact effects of VEGF on the promotion of HCC stemness are still unclear. Recently, CD133^+^, VEGFR2^+^ cells were identified as endothelial progenitor cells^14^. VEGFR2 is also preferentially expressed on the cell surface of CD133^+^ human glioma CSCs, whose viability, self-renewal, and tumourigenicity rely, at least in part, on signalling through the VEGF-VEGFR2–Neuropilin-1 axis^15^. These data suggest that CD133 and the VEGF/VEGFR2 pathway cooperate to regulate stemness, angiogenesis and tumourigenesis.

Here, we uncover a new role of VEGF in the acceleration of HCC recurrence via induction of CD133^+^ CSCs development.

## Materials and Methods

### Patient characteristics, RFA treatment and sample collection

**Figure 1:**
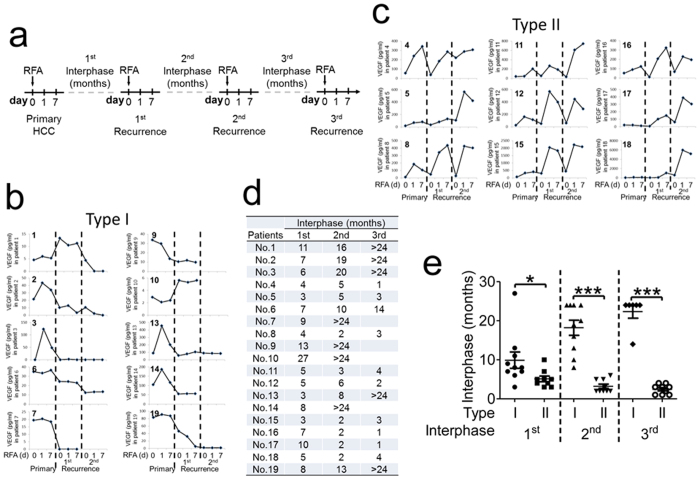
RFA-induced plasma VEGF is associated with early HCC recurrence. (**a**) A diagram of the sample collection procedure. Nineteen primary HCC patients were recruited. Before (day 0) and 1 and 7 days after RFA (day 1 and day 7, respectively), plasma and HCC tissues were collected. Most patients were treated with three RFAs, except patients 7, 9, 10 and 14, who received two treatments. RFA: radiofrequency ablation. (**b** and **c**) VEGF levels in plasma collected from patients. VEGF was measured using the Luminex cytokine assay at the indicated time points. According to the level of plasma VEGF, patients were divided into two groups: Type I (**b**) and type II (**c**). (**d**) Time to HCC recurrence following RFA in individual patients. (**e**) The mean interval for HCC recurrence was compared between type I and II patients. The values are the mean ± SEM. Note: if the interval was “>24”, “24” was used for the calculation. *p < 0.05; ***p < 0.001.

Nineteen primary HCC patients were recruited from Beijing Youan Hospital, Capital Medical University. The clinical and histological diagnoses of HCC in these patients were in accord with the diagnostic criteria recommended by the World Health Organization. Detailed patient information is shown in supplementary Table 1. Plasma was collected from the patients on day 0 (before RFA treatment) and 1 (day 1) and 7 (day 7) days post RFA treatment. When HCC recurrence was observed, RFA was administered again, and plasma was also collected on days 0, 1 and 7. Before the first RFA and at each HCC recurrence, HCC tissues were collected. Plasma and HCC tissues were stored at −80 °C and in liquid nitrogen, respectively. Detailed information regarding sample collection is shown in Fig. 1a.

### Luminex cytokine and flow cytometry assays

Plasma VEGF was detected by the Luminex cytokine assay, as previously described^16^. Flow cytometry was used to detect the number of CD133^+^ cells, as previously described^16^.

### Cell culture, shRNA-mediated interference and treatment

Human primary HCC cells (pHCCs) were isolated from specimens obtained from two HCC patients (#1 and #2) undergoing hepatic resections. Specimens used for isolating pHCCs were obtained for pathological examination; therefore, there was no need for the patient to sign an informed consent form. pHCCs were isolated from prewashed livers using a two-step collagenase perfusion and were cultivated within two layers of rat-tail collagen^17^. An HCC cell line, HepG2, was purchased from the Type Culture Collection of the Chinese Academy of Sciences, Shanghai, China. This cell line is routinely authenticated by the company, and upon receipt, no further authentication was conducted. HepG2 cells were expanded and frozen and were not passaged for more than 6 months after resuscitation. HepG2 cells were cultured as described previously^17^. shRNA-mediated interference was used to knockdown Nanog and VEGFR2 expression in HepG2 cells and pHCCs as previously described^16^. Briefly, cells were transfected with lentiviral constructs expressing shRNAs for Nanog, VEGFR2 (OriGene, MD, USA) or sh Ctrl for 24 hours. Positive cells were selected with puromycin for 7 days. Recombinant VEGF (100 ng/ml) was used to stimulate HepG2 cells or pHCCs for 72 hours. A neutralising antibody against VEGF (1 μg/ml, Abcam) and inhibitors of VEGFR1 (0.5 nmol/L, Tocris Bioscience) and VEGFR2 (100 nmol/L, Millipore) were used to inhibit protein function.

### Immunoblot assays and real-time PCR

The TaqMan method was used to quantify mRNA levels. Cluster 3.0 and TreeView were used to produce heatmaps showing the up-regulation or down-regulation of mRNA at each HCC recurrence, as previously described^17^. The details are shown in the supplementary data.

### Immunofluorescence and sphere formation assays

The details for staining CD133, Nanog, and p-VEGFR2 are shown in the supplementary data. A human CD133 MicroBead Kit (Miltenyi Biotec) was used to isolate CD133^+^ cells from HepG2 cells and pHCCs. These CD133^+^ cells were used to detect sphere formation. The details are provided in the supplementary data.

### *In vivo* assay and extreme limiting dilution analysis (ELDA)

CD133^+^ CSCs were isolated from pHCCs (#1 and #2) and were grown *in vitro* with or without VEGF stimulus. Male nude mice (strain BALB/c nude; Beijing) received subcutaneous injection of these CD133^+^ CSCs with or without VEGF stimulus (3 × 10^3^ cells/mouse, n = 10 mice per group). After 120 days, tumour sizes were measured. For ELDA assay, different dilutions (50, 100, 300, 3000, or 30000 cells/mouse) of wild type pHCCs and pHCCs with Nanog/VEGFR2 knockdown with or without VEGF pre-treatment for 72 hours (n = 10 for each group) were subcutaneously injected into nude mice to estimate the relative frequency of tumour propagating cells. After 60 days following injection, the number of nude mice with tumours was counted. An online software was used for the ELDA assay (http://bioinf.wehi.edu.au/software/elda/).

### Statistical analysis

All data shown are the results of at least three independent experiments and are expressed as the mean ± SEM. The differences between groups were compared using Student’s t-test. Differences were considered significant at confidence levels of p < 0.05 (*), p < 0.01 (**) and p < 0.001 (***), as indicated. GraphPad Prism 5 Software was used for the generation of the graphs and statistical analysis. The detailed statistical method for ELDA was described previously^18^. Briefly, the statistical p value of ELDA was obtained using a Chi-squared test.

### Study approval

The mouse-related experiments were performed in accordance with a protocol approved by the animal care and use committee of Capital Medical University affiliated Beijing You An Hospital. All animal experiments were performed according to the guidelines and approval of the institutional animal care committee. The protocol for obtaining human samples was reviewed and approved by the Ethical Review Committee of Capital Medical University affiliated Beijing You An Hospital. In addition, all methods for treating and detecting human samples were performed in accordance with the relevant guidelines of the Ethical Review Committee of Capital Medical University affiliated Beijing You An Hospital. All clinical samples were collected at Beijing You An Hospital, and informed consents were also signed.

## Results

### HCC patients with increased plasma VEGF after RFA are prone to tumour recurrence

In this study, we enrolled 19 primary HCC patients who were treated by RFA (supplementary Table 1). Plasma was collected before (day 0) and at 1 (day 1) and 7 (day 7) days after each RFA treatment (Fig. 1a). According to the level of plasma VEGF, these patients could be divided into two types: type I and II. Type I patients (n = 10) underwent a total of 25 rounds of RFA. In this group, patients 2, 3, 13 and 14 exhibited elevated VEGF at days 1 and 7 following the first round of treatment, subsequent rounds of RFA decreased or had no effect on the level of plasma VEGF (Fig. 1b). Except patients 2, 3, 13 and 14, each round of RFA decreased plasma VEGF or even had no effect on its level in the other type I patients (Fig. 1b). Moreover, for some type I patients (e.g., patient 3), plasma VEGF could not be detected even after the second or third RFA (Fig. 1b). Thus, repeated RFA decreases plasma VEGF or does not affect its level in type I patients. Type II patients (n = 9) underwent a total of 27 rounds of RFA. All type II patients except patients 17 and 18 had increased the plasma VEGF levels at days 1 and 7 following each round of RFA (Fig. 1c). Although the first RFA did not increase the plasma VEGF levels in patients 17 and 18, the VEGF levels increased after the second and third RFA in both patients (Fig. 1c). The fold increase in VEGF expression varied in this group. For example, for patient 5, after the first RFA the level of plasma VEGF on day 1 was 4-fold greater than the level on day 0; however, for patient 11, the plasma VEGF level was elevated 24-fold by day 7 relative to day 0 after the third RFA (Fig. 1c). Thus, RFA increases the level of plasma VEGF in type II patients, although different patients exhibited different increases in VEGF level. Figure 1d shows the interval for the development of HCC recurrence of each patient. By comparing the mean interval of recurrence in type I and type II patients, we found that type II patients had a shorter interval for HCC recurrence than type I patients (Fig. 1e). These data suggest that RFA-increased VEGF is associated with early HCC recurrence.

### CD133 expression is increased in recurrent HCC tissue of Type II patients after RFA

**Figure 2:**
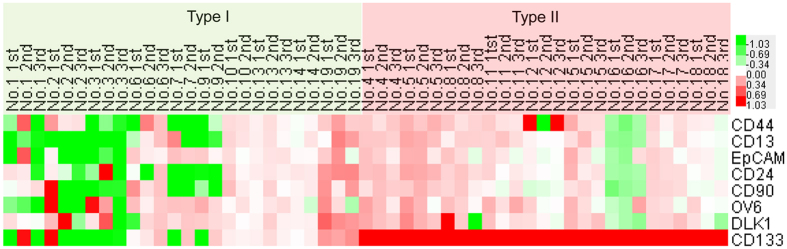
Detection the effect of RFA on the fold-change of CSCs markers in HCC tissue of type I and II patients. The TaqMan method was used to detect the mRNA levels of CSCs markers (CD13, CD24, CD44, CD90, CD133, DLK1, EpCAM and OV6) in HCC tissues of type I and II patients before and after RFA. A heatmap was used to show the up-regulation or down-regulation of CSCs markers in recurrent HCC tissue at each time point after RFA. 1st, 2nd, 3rd: 1^st^/2^nd^/3^rd^ recurrence.

We employed real-time PCR to detect the mRNA levels of CSCs markers in HCC tissue of each patient before and after RFA. The CSCs markers CD13, CD24, CD44, CD90, CD133, DLK1, EpCAM and OV6 were increased in most type II patients after RFA in comparison with type I patients, and RFA had the greatest effect on inducing CD133 expression (Fig. 2). The expression of CD133 is increased in all HCC tissue of type II patients, and its expression is the highest in comparison with the other CSCs markers (Fig. 2). These data suggest that, at least in our experimental setting, CD133^+^ CSCs are the main subpopulation of CSCs induced by RFA.

### VEGFR2 and Nanog are required for VEGF to enhance the stemness of CD133^+^ CSCs derived from HepG2 cells

**Figure 3:**
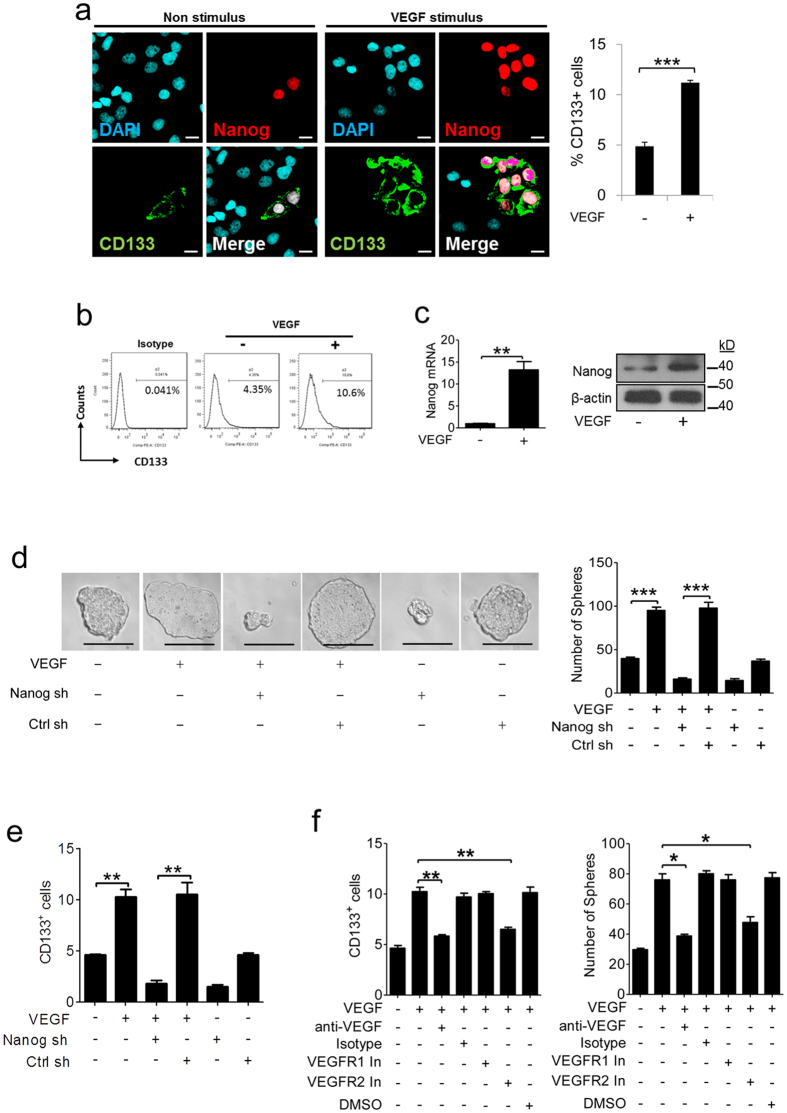
VEGF stimulus increases the level of CD133^+^ CSCs in a Nanog and VEGFR2 dependent manner. HepG2 cells were treated by recombinant VEGF for 72 hours. (**a**) A representative immunofluorescence image showing staining for CD133 (green) and Nanog (red) in HepG2 cells with or without VEGF stimulus (left panel). Scale bars = 10 μm. The levels of CD133^+^ HepG2 cells are shown as the mean ± SEM of triplicates (right panel). (**b**) Detection of CD133^+^ cells by flow cytometry. (**c**) The TaqMan method (left panel) and immunoblot analysis (right panel) were used to detect the mRNA and protein levels of Nanog in HepG2 cells with or without VEGF stimulus. (**d**) shRNA (sh)-mediated interference was used to produce a Nanog knockdown cell line in HepG2 cells. Control shRNA (Ctrl sh) was used as a knockdown control. CD133^+^ CSCs were isolated from HepG2 cells with or without VEGF stimulus, and then a sphere formation assay was used to measure the self-renewal ability of these cells. Representative images of spheres (left panel). Scale bars = 200 μm. The spheres number is shown as the mean ± SEM of triplicates (right panel). (**e**) Measuring the number of CD133^+^ CSCs derived from HepG2 cells with or without Nanog knockdown by flow cytometry. (**f**) HepG2 cells stimulated by VEGF for 72 hours with or without pre-treatment using a VEGF neutralizing antibody (anti-VEGF) or VEGFR1/2 inhibitor (VEGFR1 In or VEGFR2 In). CD133^+^ CSCs were counted by flow cytometry (left panel). The self-renewal ability of CD133^+^ CSCs isolated from HepG2 cells was assayed by sphere formation (right panel). (**e** and **f**) The values are shown as the mean ± SEM of triplicates. *p < 0.05; **p < 0.01; ***p < 0.001.

Recombinant VEGF was used to stimulate HepG2 cells for 72 hours. Immunofluorescence and flow cytometry assays showed that VEGF stimulus significantly increased the level of CD133^+^ CSCs (Fig. 3a,b). Immunofluorescence further showed that most CD133^+^ CSCs exhibit nuclear expression of Nanog (Fig. 3a). Real-time PCR and immunoblot assays also showed that VEGF stimulus induced Nanog expression (Fig. 3c). Sphere formation assays were used to measure the self-renewal capacity of CD133^+^ CSCs isolated from HepG2 cells with or without VEGF stimulation. We determined that VEGF-stimulated CD133^+^ CSCs formed more spheres than non-stimulated cells, suggesting that VEGF can enhance the self-renewal ability of CD133^+^ CSCs (Fig. 3d). shRNA-mediated interference was used to knockdown Nanog expression in HepG2 cells (Figure S1a). Nanog knockdown significantly reduced the levels of CD133^+^ CSCs and CD133^+^ CSCs-formed spheres in HepG2 cells regardless of VEGF stimulus (Fig. 3d,e), suggesting that Nanog expression is required for maintaining the size of the CD133^+^ CSCs pool and their self-renewal capacity.

VEGF receptors (VEGFR1 and VEGFR2) mediate many cellular responses to VEGF^12^. A VEGF-neutralizing antibody, VEGFR1 inhibitor and VEGFR2 inhibitor were used to disturb the interaction between VEGF and VEGFR1/2. Pre-treatment with the neutralizing antibody and VEGFR2 inhibitor significantly inhibited the ability of VEGF to induce CD133^+^ CSCs and CD133^+^ CSCs-formed spheres derived from HepG2 cells (Fig. 3f). Taken together, these data indicate that the interaction between VEGF and VEGFR2 induces Nanog expression and then contributes to the development of CD133^+^ CSCs.

### VEGFR2 is critical for enhancing CD133^+^ CSCs and promoting Nanog expression in pHCCs stimulated by VEGF

**Figure 4:**
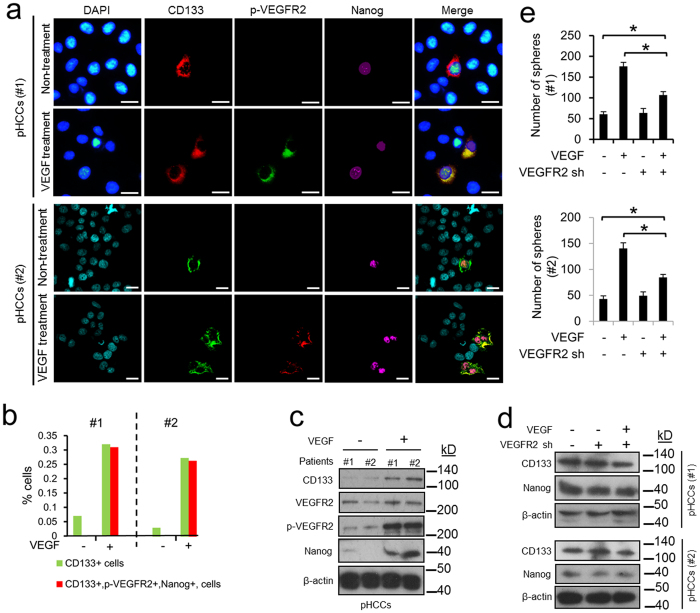
The role of VEGFR2 in VEGF-induced tumour stemness in pHCCs. Two pHCCs were isolated from two HCC patients (#1 and #2) and then stimulated by VEGF for 72 hours. (**a**) Representative immunofluorescence images showing the expression of CD133, p-VEGFR2 and Nanog in two pHCCs (#1 and #2). Scale bars = 10 μm. For pHCCs #1, red and green secondary antibodies were used to detect CD133 and p-VEGFR2, respectively. For pHCC #2, green and red secondary antibodies were used to detect CD133 and p-VEGFR2, respectively. (**b**) The levels of CD133^+^ CSCs and CD133^+^, p-VEGFR2^+^, Nanog^+^ CSCs in (**a**). (**c**) Immunoblot detection of CD133, VEGFR2, p-VEGFR2 and Nanog in two pHCCs (#1 and #2). shRNA (sh)-mediated interference was used to produce two VEGFR2 knockdown cell line from the two pHCCs. The efficiency of the knockdown is shown in supplementary Figure 1b. (**d**) Immunoblot assay demonstrating the effect of VEGF stimulus on the levels of CD133 and Nanog in two pHCCs with or without VEGFR2 knockdown. (**e**) Spheres formation assays demonstrating the effect of VEGF stimulus on self-renewal ability of pHCCs with or without VEGFR2 knockdown. The values are shown as the mean ± SEM of triplicates. *p < 0.05.

Here, we tested the effect of VEGF on activating VEGFR2 and promoting Nanog expression in two pHCCs (#1 and #2). Immunofluorescence showed that almost all CD133^+^ cells in both pHCCs expressed nuclear Nanog but were negative for p-VEGFR2 (Fig. 4a,b). In both pHCCs, VEGF stimulus increased the level of CD133^+^ CSCs, and these CSCs were Nanog and p-VEGFR2 positive (Fig. 4a,b). VEGF stimulus increased the expression of CD133 and Nanog and activated VEGFR2 as shown by the increased level of p-VEGFR2 in two pHCCs (Fig. 4c, Supplementary Figure 2). We derived VEGFR2 knockdown cell lines from the two pHCCs using shRNA-mediated interference (Figure S1b). In both knockdown lines, basal levels of CD133 and Nanog were maintained, but VEGF stimulus failed to increase the expression of CD133 and Nanog, (Fig. 4d, Supplementary Figure 3). Moreover, VEGFR2 knockdown significantly impaired the effect of VEGF on enhancing the self-renewal ability of CD133^+^ CSCs isolated from the two pHCCs, but had no effect on the basal self-renewal ability of these CSCs (Fig. 4e). Thus, VEGF increased the population of CD133^+^ CSCs via activation of VEGFR2 and induction of Nanog expression.

### CD133^+^, Nanog^+^, and p-VEGFR2^+^ cells can be identified in recurrent HCC tissue from type II patients but not type I patients after RFA

**Figure 5:**
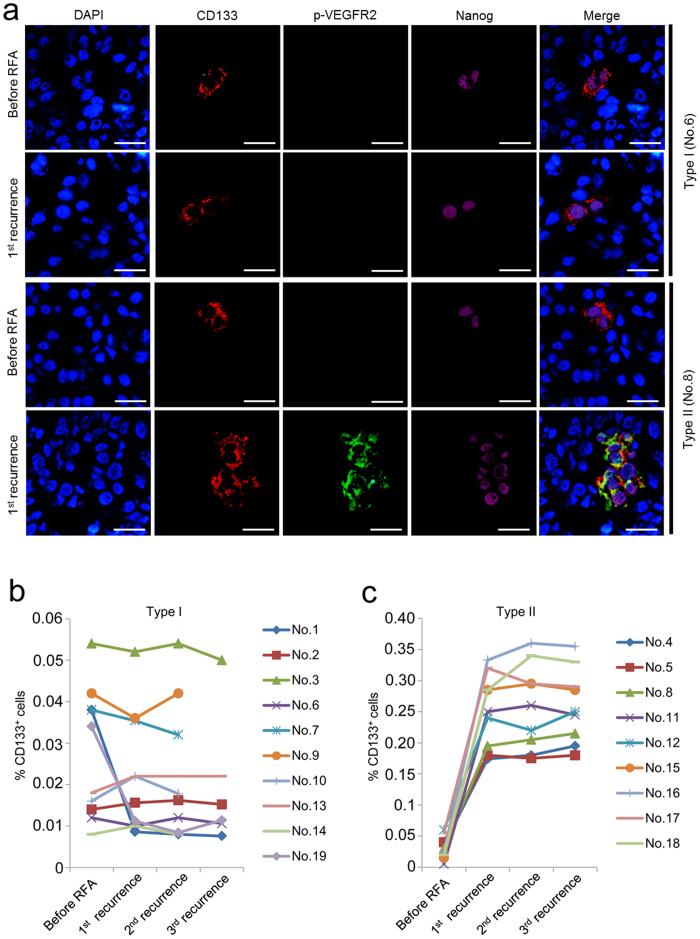
The expression of CD133, p-VEGFR2 and Nanog in HCC tissues from type I and II patients. (**a**) Representative immunofluorescence images for staining of CD133, p-VEGFR2 and Nanog in HCC tissue from patient No. 6 (type I) and No. 8 (type II) after the first RFA. (**b**,**c**) The number of CD133^+^ cells in HCC tissue of each patient at the indicated time point.

Since induction of Nanog expression and activation of VEGFR2 are required for VEGF to induce tumour stemness in HCC, we measured the expression of CD133, Nanog and p-VEGFR2 in HCC tissues from type I and II patients. In both type I and II patients before RFA, CD133^+^ CSCs were Nanog positive and p-VEGFR2 negative (Fig. 5a). In all type II patients at each recurrence after RFA, the majority of CD133^+^ CSCs were Nanog and p-VEGFR2 positive, but CD133^+^, Nanog^+^, p-VEGFR2^+^ cells could not be detected in any type I patient (Fig. 5a). Thus, we used the fold-change of CD133^+^ CSCs after RFA to evaluate the effect of this treatment on inducing tumour stemness in recurrent HCC tissues from type I and II. In 8 type I patients, RFA had no effect on the level of CD133^+^ cells; but in patients No. 1 and No. 19, RFA down-regulated the level of CD133^+^ cells by approximately 4-fold (Fig. 5b). RFA up-regulated the level of CD133^+^ cells by approximately 3- to 7-fold in type II patients (Fig. 5c). These data suggest that VEGF-induced CD133^+^, Nanog^+^, p-VEGFR2^+^ CSCs might accelerate HCC recurrence.

### VEGF pre-treatment increases the ability of CD133^+^ CSCs to form tumours *in vivo* and increases the tumorigenic cell frequency of pHCCs in a Nanog- and VEGFR2-dependent manner

**Figure 6:**
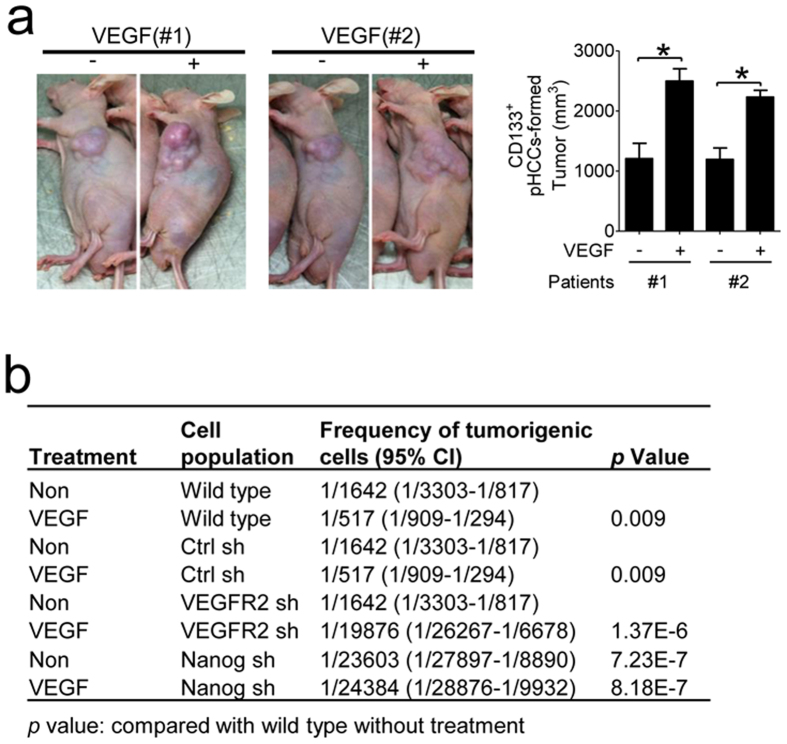
The effect of VEGF pre-treatment on the tumourigenesis of CD133^+^ CSCs and tumorigenic cell frequency *in vivo*. (**a**) CD133^+^ CSCs isolated from two pHCCs (#1 and #2) with or without VEGF pre-treatment were subcutaneously injected into nude mice (3 × 10^3^/mice). The tumour size was measured after growing for 120 days *in vivo* (right panel, n = 10 for each group). Representative images of xenograft tumour (left panel). (**b**) shRNA (sh)-mediated interference was used to knockdown Nanog and VEGFR2 in pHCCs (#1). The effectiveness of the knockdown is shown in supplementary Figure 1b and 1c. Wild type, Nanog knockdown or VEGFR2 knockdown pHCCs were treated with VEGF for 72 hours and were then subcutaneously injected into nude mice (50 ~ 3 × 10^4^/mice). The tumorigenic cell frequency was analysed with a limiting dilution assay (n = 10 for each group).

CD133^+^ CSCs were isolated from pHCCs of two HCC patients (#1 and #2) with or without VEGF pre-treatment and then these CSCs were transplanted subcutaneously into nude mice (3 × 10^3^ CD133^+^ CSCs/mice). We discovered that VEGF-pre-treated CD133^+^ CSCs formed larger xenograft tumours than non-treated CSCs (Fig. 6a). Then, we used extreme limiting dilution analysis to evaluate the effects of VEGFR2 and Naong on the tumourigenic cell frequency of pHCCs with or without VEGF pre-treatment. pHCCs (#1) with knockdown of VEGFR2 or Nanog were cultured with or without recombinant VEGF and were then transplanted subcutaneously into nude mice (from 50 to 3 × 10^4^ CD133^+^ CSCs/mice). VEGF pre-treatment significantly increased the tumourigenic cell frequency of cultured pHCCs relative to the frequency obtained from the non-treated controls (Fig. 6b). VEGFR2 knockdown significantly reduced the tumourigenic cell frequency of pHCCs pre-treated with VEGF but had no effect on the basal tumourigenic cell frequency (Fig. 6b). Nanog knockdown significantly reduced the tumourigenic cell frequency regardless of VEGF stimulus (Fig. 6b). Thus, our data demonstrate that VEGF signalling via VEGFR2 and Nanog plays a vital role in tumor stemness in HCC following RFA.

## Discussion

The role of VEGF in angiogenesis and lymphangiogenesis has dominated the VEGF-related research field, with much of the literature being devoted to elucidating the molecular mechanisms regulating these processes^19^,^20^. However, the function of VEGF is not limited to angiogenesis and vascular permeability^21^. Autocrine and paracrine VEGF signalling occurs in some tumour cells, contributing to induction of CSCs, independent of any role that VEGF may have in angiogenesis^12^,^22^. CSCs are localized in a perivascular niche, and VEGF secreted from these cells functions in a paracrine manner to stimulate angiogenesis in nascent tumours; simultaneously, autocrine VEGF signalling can promote dedifferentiation and an epithelial-mesenchymal transition (EMT) resulting in increased migration and invasion into the stroma^12^. One study demonstrated that tumours up to 24 hours after RFA treatment exhibit a region of tumour necrosis (zone of white coagulation) surrounded by a region exhibiting vascularization and early inflammatory markers^23^. RFA-induced microvascular and tissue injury promotes angiogenesis to relieve ischaemia in local areas^23^, and this VEGF-induced angiogenesis may act synergistically with VEGF-induced tumour stemness to accelerate HCC recurrence, as shown by our results that VEGF signalling enhances the tumourigenicity of CD133^+^ CSCs isolated from patients with an increased risk for early recurrence. Additional studies have shown that increased VEGF or an activation of VEGF signalling after RFA is associated with a worse prognosis in HCC^24^,^25^,^26^. Our study further suggests that the function of CSCs on inducing tumorigenesis is one of the reasons why activation of VEGF signalling is associated with poor survival or outcome in HCC.

How might VEGF induce tumour stemness in HCC? To our knowledge, this question remains unanswered. It is known that VEGF receptor tyrosine kinases (RTKs) (VEGFR1, VEGFR2) and neuropilins (NRPs) are involved in VEGF-mediated tumour stemness. VEGFR2 is the predominant receptor tyrosine kinase (RTK) that mediates VEGF-regulated angiogenesis^27^. Our study demonstrated that VEGFR2, but not VEGFR1, transduces signals that control stemness. Similarly, other studies have shown that VEGFR2 regulates stemness in cancer cells^13^,^15^,^28^. Recently, NRPs were also shown to control VEGF-induced tumour stemness^12^. Our data showed that knockdown of NRPs via shRNA could not affect the size of the CD133^+^ CSCs pool in HepG2 cells and in two pHCCs with or without VEGF stimulus (Supplementary Figure 4). However, NRPs form complexes with VEGFR1 and VEGFR2, enhancing the affinity of these receptors for VEGF^29^; and in some tumour cells expressing VEGFR1 in the absence of VEGFR2, VEGFR1 seems to use its RTK as a receptor to transduce VEGF signalling. To comprehensively elucidate how receptors of VEGF mediate VEGF-induced tumour stemness in HCC recurrence, additional pHCCs and other cell lines should be used in the future.

Vasculogenic mimicry (VM) is important for the growth and progression of tumours^30^, and CSCs are known to induce VM development. For example, CD133^+^ CSCs are localized in perivascular niches and they induce the formation of VM in melanomas^31^. VM is thought to enhance the supply of blood during the early stages of tumour growth; however, during tumour progression, the role of VM is reduced and the number of endothelial-dependent vessels increased^32^. Thus, our data suggest that CSCs-induced VM might play a role in accelerating the recurrence of HCC after RFA by increasing blood supply. However, the real role of VEGF-induced CSCs on inducing VM formation after RFA must still be tested using more clinical samples in the future.

In this study, we have shown that Nanog is required for maintaining the size of the CD133^+^ CSCs pool regardless of VEGF stimulus. Nanog is induced generally during very early embryonic stages and in germline stem cells, which is required for the pluripotency of embryonic stem cells (ESCs)^33^. Nanog is not expressed in normal somatic tissue but is present in many cancers, including HCC, where its expression is associated with a poor prognosis^34^. Our data reveal that VEGFR2 can stimulate Nanog expression in pHCCs, although the intermediate steps in this signalling process remain unclear. It is known that VEGFR2 can stimulate the PI3K/AKT pathway^35^,^36^, and activated AKT is involved in the regulation of Nanog expression^37^. Thus, future studies should test whether the VEGFR2/PI3K/AKT pathway induces Nanog expression in HCC CSCs.

Taken together, our data have revealed a mechanism that can explain why some RFA-treated HCC patients are prone to suffer early recurrence. We believe our data (e.g., the level of plasma VEGF) are beneficial for the prediction of HCC recurrence after RFA.

## Additional Information

**How to cite this article**: Liu, K. *et al*. CD133^+^ cancer stem cells promoted by VEGF accelerate the recurrence of hepatocellular carcinoma. *Sci. Rep.* **7**, 41499; doi: 10.1038/srep41499 (2017).

**Publisher's note:** Springer Nature remains neutral with regard to jurisdictional claims in published maps and institutional affiliations.

## Supplementary Material

Supplementary Data
