# Supplementary material for: CD133+ cancer stem cells promoted by VEGF accelerate the recurrence of hepatocellular carcinoma
Source: Sci Rep. 2017 Jan 30;7:41499. doi: 10.1038/srep41499 (PMC5278354; doi:10.1038/srep41499)
Supplement: Supplementary Data [file srep41499-s1.doc]

**Supplementary Data**

**Title page**

**Title:** CD133^+^ cancer stem cells promoted by VEGF accelerate the recurrence of hepatocellular carcinoma

**Running Title:** VEGF induces tumor stemness in HCC

**Authors:** Kai Liu^1,2,*^, Meijun Hao^1,*^,Yabo Ouyang^1,2^, Jiasheng Zheng^1,§^, Dexi Chen^1,2,3,§^

**Affiliations and addresses:** 1. Capital Medical University affiliated Beijing You An Hospital, Beijing, 100069, China; 2. Beijing Institute of Hepatology, Beijing, 100069, China; 3. Organ Transplantation Center, the Affiliated Hospital of Qingdao University, Qingdao City, Shandong Province, 266003, China

* The authors contributed equally to this work.

^§^ Corresponding author

**^#^Correspondence:** Dr. Dexi Chen and Prof. Jiasheng Zheng, Address: No. 8 XiTouTiao, YouAnMenWai Street, FengTai District, Beijing 100069, P.R. China. Telephone: +8610-63057109, Fax: +8610-63057109, E-mail: dexi0963@yahoo.com; jiashengzheng@outlook.com .

**Keywords:** HCC; CSCs; VEGF; CD133

**Conflicts of interest**: The authors disclose no conflicts.

**Acknowledgements:** This work was supported by National Natural Science Foundation of China (NSFC 81402556, 81272266), National Key Technology R&D Program (2012BAI15B08), International Cooperation and Exchanges NSFC (81361120401)，and Capital Health Research and Development Special grants (2014-1-1151).

**Contributions:** Dexi Chen and Jiasheng Zheng designed all the experiments in this study and guided Kai Liu to complete the writing. Kai Liu completed almost all detections *in vitro* and *in vivo*, analyzed all the experiments-derived data and completed the writing. Meijun Hao collected all the samples of HCC patients treated by RFA and provided their clinical information. Yabo Ouyang assisted Kai Liu to complete some immunoblotting detection.

Supplementary table 1. The clinical information of HCC patients


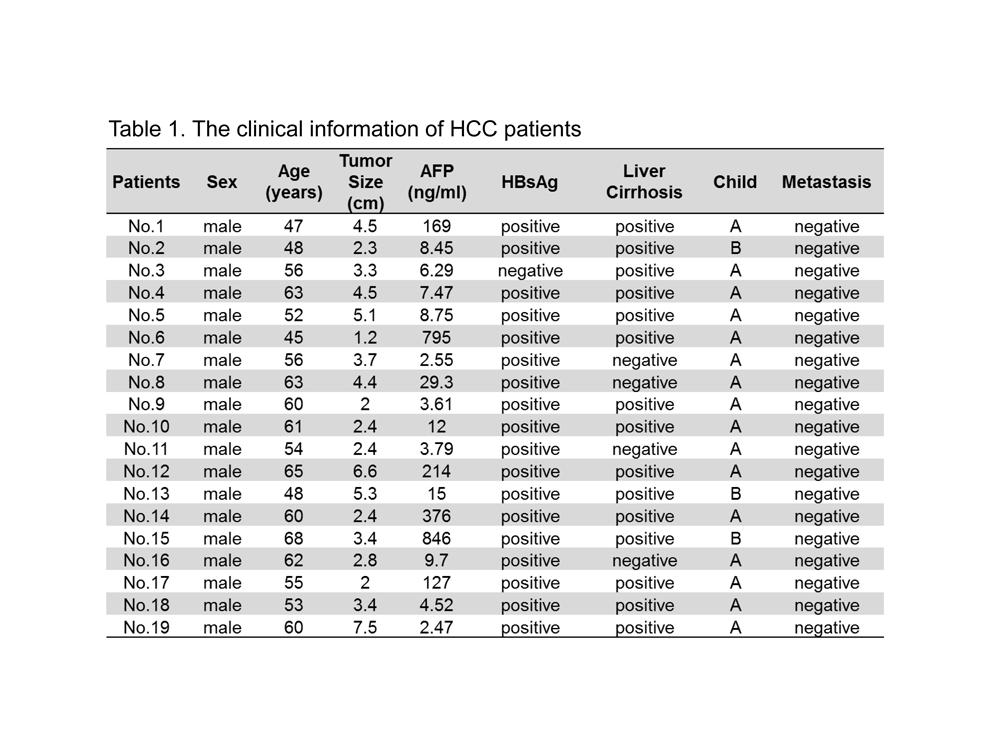


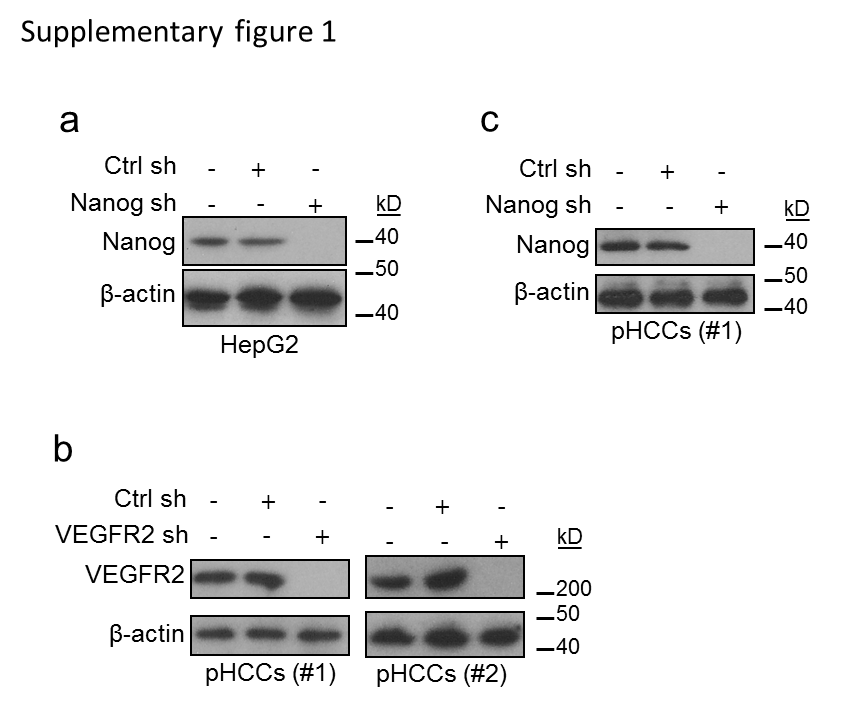


**Figure S1**. **Immunoblot detection the effect of shRNA-mediated knockdown of Nanog and VEGFR2.** (a, c) Immunoblot detection the expression of Nanog in HepG2 cells (a) and pHCCs (#1), which stably expressing Nanog shRNA (c). (b) Immunoblot detection the expression of VEGFR2 in two pHCCs (#1 and #2), which stably expressing VEGFR2 shRNA.


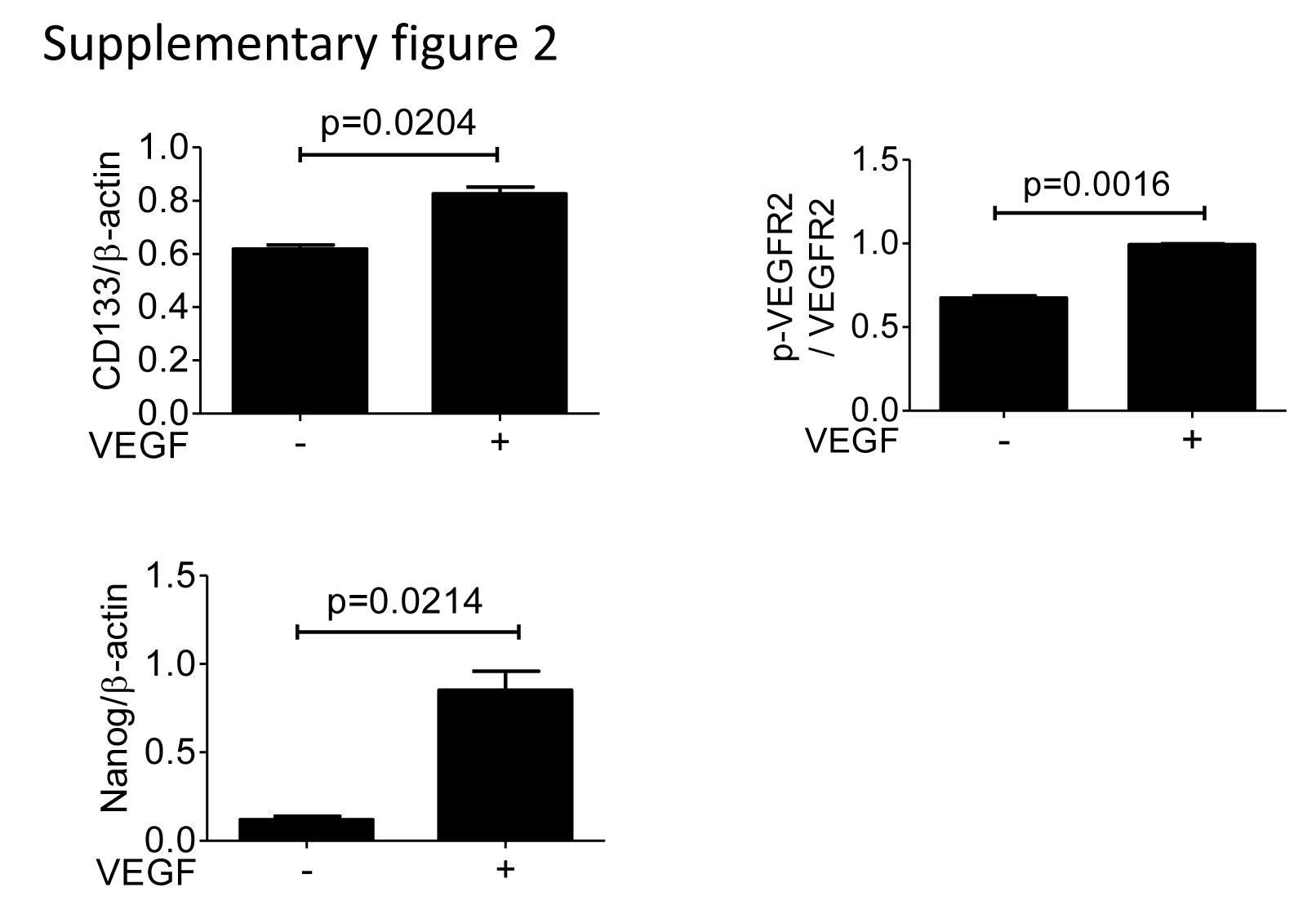


**Figure S2. Densitometry analysis of the band density ratio of CD133/Nanog to β-actin and p-VEGFR2 to VEGFR2 of figure 4c.** The grey values were obtained by scanning the bands of CD133, Nanog, β-actin, p-VEGFR2 and VEGFR2 of pHCCs #1 and #2 in figure 4c.


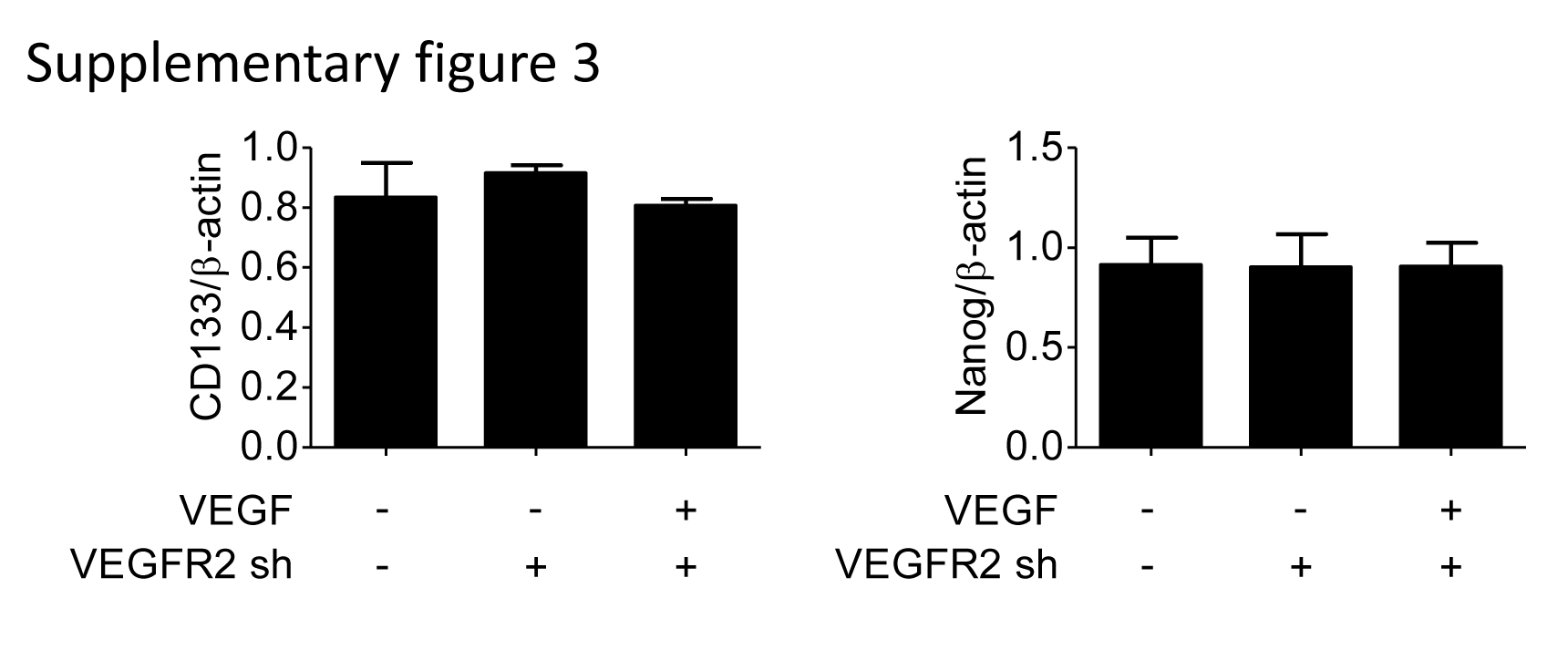


**Figure S3. Densitometry analysis of the band density ratio of CD133/Nanog to β-actin of figure 4d.** The grey values were obtained by scanning the bands of CD133/Nanog/β-actin of pHCCs #1 and #2 in figure 4d.


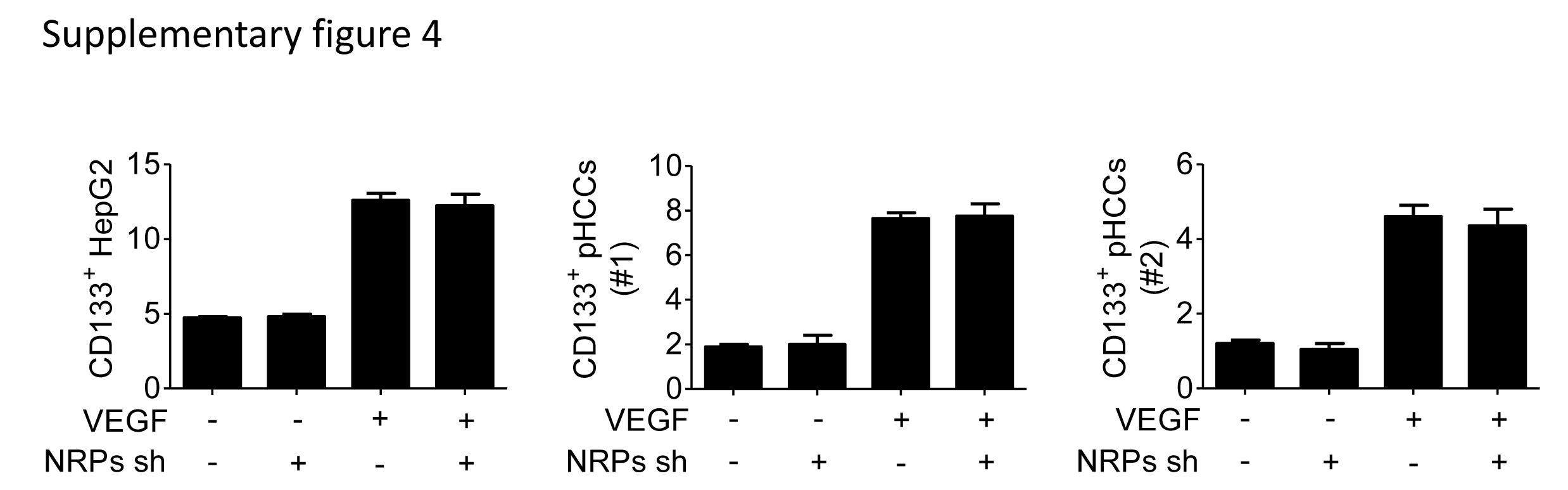


**Figure S4. Knockdown of NRPs via shRNA did not affect the levels of CD133^+^ CSCs in HepG2, pHCCs #1 and pHCCs #2.** The expression of NRPs was knockdown by shRNA in HepG2, pHCCs #1 and pHCCs #2, and then flow cytometry were used to detect the levels of CD133^+^ cells.

**Supplemental materials and methods**

**Cell culture and treatment.** HepG2 cells were grown in Dulbecco’s modified Eagle's medium (DMEM). DMEM was supplemented with 10% fetal bovine serum (FBS). Cell lines were treated by recombinant VEGF (100ng/ml) (R&D Systems). Neutralising antibody of VEGF (1 μg/ml, Abcam), VEGFR1 inhibitor (0.5nmol/L, Tocris Bioscience) and VEGFR2 inhibitor (100nmol/L, Millipore) were used to inhibit related proteins’ function.

**Immunoblot assay and real-time PCR.** Cell lysates were subjected to immunoblot analysis and real time PCR was used to detect the mRNA level, as previously described ^1^. Anti-β-actin/Nanog came from Abcam; anti-VEGFR2/p-VEGFR2/CD133 came from Cell signaling. The mRNA content was normalized to the expression of the housekeeping gene β-actin. The sequences of β-actin primers are described in reference ^1^. The mRNA of CD13, CD24, CD44, CD90, CD133, DLK1, EpCAM, OV6 and Nanog were detected by Taqman method, the primers and probes come from lifetechnologies.

**Cluster and treeview**. Total RNA was isolated from HCC tissue before RFA and at each recurrence. Real time PCR (Taqman method) was used to detect the levels of CD13, CD24, CD44, CD90, CD133, DLK1, EpCAM and OV6. Compared with the level of mRNA before RFA, is represented by 2^-ΔΔC^_T_ value. The 2^-ΔΔC^_T_ value of each gene was analyzed by cluster 3.0 and heatmap is produced by TreeView ^2^.

**Immunofluorescence Assay.** Immunofluorescence staining for CD133, Nanog, p-VEGFR2 were performed as described previously ^1^. Immunofluorescence images were detected using a fluorescence microscope (Nikon Eclipse 80i).

**Sphere formation assay**. Human CD133 MicroBead Kit (Miltenyi Biotec) was used to isolate CD133^+^ cells from HepG2 cells and pHCCs. CD133^+^ cells were suspended in serum-free DMEM/F12 supplemented with 100 IU/ml penicillin, 100 μg/ml streptomycin, 20 ng/ml human EGF, 10 ng/ml human FGF, 2% B27 supplement without vitamin A, 1% N2 supplement (Life Technologies) ^3^. The cells were subsequently cultured in ultra low attachment plates (Corning Inc) for 72 hours.

**References**

1. Liu, K. *et al*. CHOP Mediates ASPP2-induced Autophagic Apoptosis in Hepatoma Cells by Releasing Beclin-1 From Bcl-2 and Inducing Nuclear Translocation of Bcl-2. *Cell Death Dis*. 5, e1323 (2014).

2. Liu, K. *et al*. Nuclear EGFR Impairs ASPP2-p53 Complex-Induced Apoptosis by Inducing SOS1 Expression in Hepatocellular Carcinoma. *Oncotarget*. 6, 16507-16516 (2015).

3. Cao, L. *et al*. Sphere-Forming Cell Subpopulations with Cancer Stem Cell Properties in Human Hepatoma Cell Lines. *Bmc Gastroenterol*. 11, 71 (2011).
